# Prenatal mercury exposure, autism, and developmental delay, using pharmacokinetic combination of newborn blood concentrations and questionnaire data: a case control study

**DOI:** 10.1186/s12940-015-0045-4

**Published:** 2015-07-22

**Authors:** Stephen J. McKean, Scott M. Bartell, Robin L. Hansen, Gry H. Barfod, Peter G. Green, Irva Hertz-Picciotto

**Affiliations:** University of California, Davis, Davis, CA USA; Program in Public Health and Department of Statistics, University of California, Irvine, Irvine, CA USA; Department of Pediatrics, University of California, Davis, Sacramento, CA USA; MIND Institute, School of Medicine, University of California, Davis, Sacramento, CA USA; Department of Civil and Environmental Engineering, University of California, Davis, Davis, CA USA; Department of Public Health Sciences, University of California, Davis, Davis, CA USA; Interdisciplinary Center for Plasma Mass Spectrometry, University of California, Davis, Davis, CA 95616 USA

## Abstract

**Background:**

Methylmercury (MeHg), known for well over a century as a neurotoxin in adults, has more recently been studied for potential detrimental effects during early brain development. While several studies have estimated mercury exposure, they usually rely on either a single biomarker or questionnaire data, each of which has limitations. The goal of this paper was to develop a toxicokinetic model that incorporates both biomarker and questionnaire data to estimate the cumulative exposure to MeHg through seafood consumption using data collected from the Childhood Autism Risks from Genetics and the Environment (CHARGE) study.

**Methods:**

We utilized a previously described discrete-time model that estimates blood MeHg concentration given a piecewise-constant ingestion rate and single-compartment pharmacokinetics. We measured newborn bloodspot Hg concentrations and obtained information pertaining to maternal fish consumption using a questionnaire. Using MeHg concentration estimates from the toxicokinetic model, cumulative MeHg exposure was estimated in children with autism, children with developmental delay, and typically developing children. Median estimated cumulative MeHg was compared among diagnostic groups using the Kruskal-Wallis Test. Multinomial logistic regression models were constructed to assess the association between cumulative MeHg concentration and the risk of autism and developmental delay (vs. typical development).

**Results:**

The estimated average MeHg concentration of for all fish species consumed by mothers was 42 ppb. Median cumulative MeHg over gestation was similar across diagnostic groups (p-values raged from 0.91 to 0.98). After adjusting for potential confounding, we found no association between cumulative MeHg exposure and the risk of autism (OR = 0.95, 95 % CI: 0.95, 1.12) or developmental delay (OR = 1.00, 95 % CI: 0.89, 1.13).

**Conclusions:**

The toxicokinetic model described in this paper yielded fish MeHg concentration estimates that are consistent with fish species containing lower levels of MeHg. Overall, cumulative MeHg exposure does not appear to detectably elevate the risk of autism or developmental delay. Based on the regression standard error for the association between ASD and TD, we would have reported statistical significance for an adjusted odds ratio of 1.09 or larger. This method can easily be extended to other epidemiologic studies in which there is a biomarker measurement and questionnaire data regarding exposure.

## Background

Methylmercury (MeHg) is an environmental contaminant and a known neurotoxin [1]. Concern exists because the nervous system has been shown to be, at high doses, especially sensitive to mercury during the prenatal period [2, 3]. Additionally, because the primary exposure to MeHg is seafood, low-level exposures are common [4]. Fetal exposures occur because MeHg readily crosses the placental barrier [4]. While several studies have measured biomarker mercury concentrations to estimate exposure, they are often limited by the number and timing of measurements (typically one sample per participant). This is especially true when measuring exposure over longer durations of time (on the order of months or years, e.g. the entire duration of a pregnancy), without relying on an unrealistic but common assumption that blood mercury concentrations are at steady-state. In epidemiological studies, obtaining numerous biological samples over time is atypical due to the high cost and excessive burden on study participants. As a result, one biomarker is often measured, limiting the interpretation to the appropriate time period the biomarker represents [5, 6]. For example, one recent study found substantial variability and a poor correlation (0.19-0.63) for maternal blood mercury concentrations across trimesters [6], indicating that a single blood sample might be inadequate for assessing mercury exposure throughout pregnancy. Alternatively, food frequency questionnaires are commonly implemented to assess past toxicant exposures, including mercury. To estimate the ingested dose, this method often relies on published food toxicant concentration values external to the study [7]. This approach becomes less reliable when concentrations vary widely within or across sources of exposure, as is the case with MeHg in populations with access to a range of seafood options. Additionally, evidence suggests that long-term MeHg exposures based solely on food frequency questionnaires are overestimated, possibly resulting from the over report of fish consumption and the utilization of literature-based (versus directly measured) MeHg concentrations for food items [8–10].

Because biomarker or questionnaire data can limit the interpretability of the results when used alone, there is increased interest in developing methods that combine these two types of information in order to gain the advantages of both. The goal of this paper was to develop a toxicokinetic model to estimate the cumulative exposure to MeHg in children during the gestational period using data collected from the Childhood Autism Risks from Genetics and the Environment (CHARGE) study. CHARGE is an ongoing large-scale case–control study focusing on several genetic and environmental exposures as underlying causes of autism [11]. We utilized newborn bloodspots collected shortly after birth (which largely represent third trimester MeHg exposures) and questionnaire data regarding fish consumption shortly before and throughout pregnancy. We incorporated the questionnaire data, blood spot mercury concentrations, and toxicokinetic parameters into a single model to estimate the average MeHg concentration of the fish consumed by mothers in our study, effectively calibrating the exposure model to the measured blood spot concentrations. Then, using the estimated fish MeHg concentrations, we estimated the cumulative fetal dose of MeHg over the gestational period from maternal fish consumption. Estimated cumulative MeHg doses were then compared among cases and controls.

## Methods

### Study population

#### Eligibility

The CHARGE study was initiated in 2002 and is an ongoing population-based case–control study with participants from three strata: (1) children with autism spectrum disorder (ASD); (2) children with developmental delay but not autism (DD); (3) children selected from the general population without regard to developmental characteristics (GP). Eligible children met the following criteria: (a) between the ages of 24 and 60 months; (b) living with at least one biological parent; (c) has a parent who speaks English or Spanish; (d) born in California; (e) resides in the study catchment areas of California, including more than 20 counties within a 2-h drive from the MIND Institute clinic located at the UC Davis Medical Center in Sacramento.

### Sampling frame and recruitment

Children diagnosed with autism or developmental delay were identified through the California Department of Developmental Services (DDS), which, through its system of Regional Centers (RCs), coordinates services and support to individuals with developmental disabilities. Children with an autism diagnosis who did not enroll in the RC system were also eligible. The group from the general population was sampled from California state birth files. Throughout the study, random samples of children meeting study eligibility criteria according to information recorded on the birth certificate were generated. This group was frequency-matched to the projected age, gender, and Regional Center catchment area distribution of autism cases. Study personnel used parents’ names and social security numbers from the birth files to locate a current address and phone number for selected GP families. English and Spanish-speaking study personnel made up to 20 phone calls to contact each selected family and mailings were also sent.

### Diagnostic validation

Trained CHARGE and MIND Institute personnel who had attained research reliability conducted standardized clinical assessments to confirm the child’s diagnostic group. Autism cases were assessed using the Autism Diagnostic Interview-Revised (ADI-R) [12–14] and the Autism Diagnostic Observation Schedules-Generic (ADOS-G) module 1 or 2 [15]. The ADI-R is a standardized, semistructured interview administered to the caregiver, and is designed to assess impairments in reciprocal social interaction, communication, and repetitive behaviors. The ADI-R interrater reliability kappa values are between 0.62 and 0.89 [14]. The ADOS is a standardized, semistructured assessment of social interaction, and play or imaginative use of materials. Unlike the ADI-R, the ADOS requires an examiner to observe the child’s behavior. Kappa values for interrater reliability are above 0.60 [16].

Cognitive function was measured in all children using the Mullen Scales of Early Learning (MSEL) [17]. Adaptive function was assessed by parental interview using the Vineland Adaptive Behavior Scales [18]. The evaluation for the DD and GP children was completed with the same protocol as children with autism, minus the ADI-R and ADOS. All controls (DD and GP) were screened for autism spectrum disorders using the Social Communication Questionnaire (SCQ) [19]. If the SCQ score was >15, the ADI-R and ADOS were then used to evaluate for ASDs. Final autism case status (ASD) was defined as : 1. meeting criteria on the communication, social interaction, and repetitive behaviors domains of the ADI-R and scoring at or above the total cut off for autistic disorder on the ADOS module 1 or 2 ; 2. children who met the criteria on either the communication or repetitive behavior domains of the ADI-R, were within 2 points of meeting the criteria on the other domain, and met the requirements for ASD on the social and communication domains of the ADOS. The DD group is comprised of children who obtained an MSEL score of < 69 and a VABS composite score of < 70. Children from the general population who were not DD or AU were identified as typically developing (TD) if they scored: (1) 71 or above on Mullen, (2) 71 or above on Vineland and (3) <15 on SCQ. Children who were scored as typically developing according to our validation but entered the study with a diagnosis of ASD or DD were classified as atypically developing (AtD) and were combined with the DD group for the purposes of this study.

### Data collection

#### Exposure assessment and other variables

Trained bilingual and bicultural (English and Spanish) interviewers asked the primary caregiver questions over the telephone regarding peri-conceptional, prenatal, and early childhood exposures and experiences. The interview included questions about maternal fish consumption during three, two, and one month prior to pregnancy, and during each trimester. Questions distinguished among tuna (fresh or canned), other ocean fish, freshwater fish, and fish caught by the participant or by someone they knew. If the participant or someone they knew caught the fish, they were asked to specify the exact type, and we categorized it as tuna, other ocean, or fresh water. One serving of tuna was defined as 3 ounces of fresh tuna, one half can, or one fish sandwich. For each fish category, we asked about frequency of servings per week, categorized as: 0 (none), greater than zero but less than one, one, and more than one. The caretaker was provided with a list of fish species and which category they fell under. Other covariate information was collected including maternal and paternal education, maternal age, and ethnicity. Gestational age of the child was abstracted from medical records.

### Bloodspot collection and laboratory analysis

Bloodspots collected at birth for newborn screening were obtained from California state archives. The blood samples were analyzed blindly with respect to group. Total blood mercury was measured at the UC Davis Inductively Coupled Plasma-Mass Spectrometry (ICP-MS) (Agilent Technologies, Santa Clara, CA) Center (icpms.ucdavis.edu) using laser ablation with a 213 nm laser (New Wave) equipped with a SuperCell to improve Hg performance-both washout and signal strength. Blood spot samples and five standards were mounted on sterilized glass slides with double-sided adhesive tape and loaded into the laser chamber with standards prepared from clinical laboratory reference blood (Wadsworth Center, Albany, NY). For each blood spot, measurements were taken on one line scan (0.5 mm long). New Wave’s software program GLITTER was used to analyze the bloodspot mercury data. Measurements were in parts per billion, with a detection limit of 0.01 ppb. Values were imputed for bloodspot reading below the limit of detection by dividing the limit of detection by the square root of two.

### Data analysis and statistical procedures

#### Preliminary analyses

We examined the univariate distribution of the natural log bloodspot Hg concentration to ensure that the levels measured in our study are consistent with the previously reported values, bearing in mind that newborn blood Hg concentrations are higher than maternal blood Hg concentrations. Additionally, we constructed a linear regression model to verify whether fish consumption (summed across fish types) during the third trimester predicted bloodspot Hg concentration.

### Pharmacokinetic models

We utilized a previously described discrete-time model [20, 21] that estimates blood MeHg concentration given a piecewise-constant ingestion rate and single-compartment pharmacokinetics. A single-compartment model has been shown to adequately represent the accumulation and excretion of MeHg in humans [1]. In our study, we measured the blood Hg concentrations in the bloodspots and the time-varying rate of fish consumption from the questionnaire, and from this information, we wish to estimate the average mercury concentration of the fish consumed by the mothers during and just prior to pregnancy in our study. Blood mercury concentrations C_B_ (micrograms per liter) at the time of collection (tq) were modeled using the following equation:

$$ {C}_B\left({t}_q\right)={\displaystyle \sum_{j=1}^q}\frac{fc{I}_j}{k{V}_{i,j}}\left(1-{e}^{-k\left({t}_j-{t}_{j-1}\right)}\right)\left({e}^{-k\left({t}_q-{t}_j\right)}\right) $$

where I_j_ is the fish intake rate (Kg/day) during time period j, c is the fish MeHg concentration, f is the fraction of ingested MeHg remaining in the blood after it is absorbed across the gastrointestinal tract and distributed throughout the body, k is the first-order rate constant for MeHg elimination (day^−1^), and V_i,j_ is individual blood volume (in liters) during each time period. We assume that fish mercury concentration is constant over time.

The equation above can be rewritten to show that blood MeHg concentration is a function of the constants (f, k, V) and the time weighted fish consumption rate and the fish MeHg concentration c:

$$ {C}_B\left({t}_q\right)=\frac{f}{k{V}_{i,j}}{\displaystyle \sum_{j=1}^q}{\alpha}_jc{I}_j=\frac{f}{k{V}_{i,j}}c\overline{I} $$

where the weighted average of the time-varying fish consumption rate is given by:

$$ \overline{I}={\displaystyle \sum_{j=1}^q}{\alpha}_j{I}_j $$

and the weights (accounting for the elimination of mercury from the blood over time) during each time step are given by:

$$ {\alpha}_j=\left(1-{e}^{-k\left({t}_j-{t}_{j-1}\right)}\right)\left({e}^{-k\left({t}_q-{t}_j\right)}\right) $$

Fish consumption occurring further away in time prior to bloodspot collection carries less weight while fish consumption that occurred closer to bloodspot collection (e.g. the third trimester) carries more weight in terms of predicting bloodspot mercury concentration. Mercury exposures prior to 3 months preconception are assumed to have a negligible contribution to blood mercury concentrations at birth. Notably, the blood mercury concentration is expected to be a linear function of the fish mercury concentration. Background (non-fish) mercury exposures should constitute an additive contribution if those exposures are constant over time. Thus, the fish mercury concentration can be estimated using ordinary linear regression if the other parameters are known (with fish MeHg concentration acting as the estimated beta coefficient, *f Ī*/*kV* as the “covariate”, and the background MeHg contribution as the intercept).

We developed several models to estimate average fish concentration. First, we predicted the fish concentration separately for each category of fish in the CHARGE questionnaire (Tuna, Other Ocean, and Freshwater). Fish that was caught, when possible, was categorized into one of the three fish types. Two mothers ate unidentified caught fish types, and were excluded. Frequency of consumption during each time period (I_j_) was assigned the following values: 0 (none); 0.5 (more than 0 but less than 1 serving per week); 1 (1 serving per week); 2 (>1 serving per week). We also ran the regression model described above with the fish consumption rates summed across fish type, and assigned continuous values to the following categories: 0 (none), (1) between 0 and 2 servings, (3) 2 or more servings. To make the units consistent, servings per week were converted to Kg/day. We assumed an average serving size to be 6 oz or 0.17 Kg.

Time steps (t_j_, in days) were assigned based on time periods covered by the CHARGE questionnaire, with intervals of: −90 to −60 days (3 months preconception), −60 to −30 days (2 months preconception), −30 to 0 days (one month preconception), 0 to 90 days (1^st^ trimester), 90–180 days (2nd trimester), and 180 days until birth (3^rd^ trimester, using the recorded gestational age at birth for each child). For children born before the beginning of the 3rd trimester, the gestational age at birth was used to determine the upper limit for the interval associated with the 2^nd^ trimester. The MeHg elimination rate, k, was assumed to be 0.014 day-1 based on the mean value obtained in a controlled dosing study [22]. The fraction (f) of MeHg remaining in blood after absorption and distribution is approximately 0.05 [22].

Individual blood volume was estimated from maternal weight data during each time period. For some mothers, weight information was available from both medical records and through self-report, while others only had weight information from one of the sources of information. We assigned the mother’s weights for each time period based on a scheme that favored medical record data when available. For prepregnancy time periods, we assigned the medical record prepregnancy weights if they were available (52 %). If they were not available, then we assigned the self-reported values from the EEQ. For first trimester weight, we assigned the weights from the medical records for the first prenatal visit if the first prenatal visit occurred during the first trimester (gestational age ≤12 weeks). If the medical record was not available or the there was no prenatal visit during the 1st trimester, we assigned the medical record prepregnancy weight or the reported prepregnancy weight (depending on availability). For the majority of mothers, the weight is similar between the two time periods (often the same or elevated a few lbs in the first trimester versus prepregnancy). This is typical during most pregnancies [23]. For the third trimester, we assigned the medical record weight during the last prenatal visit, if this occurred during the third trimester. If this was not available, we assigned the medical record weight during the admission for delivery if admission was during the third trimester (there are only a few instances where admission weight is available, but last prenatal visit is not). If no medical record data was available for third trimester weight, then the prepregnancy weight plus the self-reported weight gain during pregnancy was used. In the few cases where child delivery occurred prior to the third trimester, the weight was assigned to the second trimester. Because we do not have data for second trimester weights, we assigned the first trimester weight plus half of the weight gained during pregnancy. These values can be reasonably interpolated in this way because weight gain is fairly linear between the first and the third trimester [23].

Blood volume was determined from maternal weight using two methods. In the first method, volume was converted from maternal weight in kilograms to blood volume in using previously estimated conversion factors. For the prepregnancy periods and the first trimester, we used 0.065 L/Kg [24]. Conversion factors during pregnancy range from 0.073 to 0.096 L/Kg [25]; we used 0.080 L/Kg for the second and third trimesters. The second method to assign blood volume relied on the increase in weight during pregnancy rather than absolute weight during each time period. Because there is no expected weight gain during the prepregnancy period and very little, if any, during the first trimester, we again assigned 0.065 L of blood per kilogram of body weight. For the second trimester and third trimesters, we assumed blood accounted for 15 % and 20 % of the total weight gained over pregnancy, respectively [23]. The proportion of weight attributed to blood was converted to liters by dividing the weight of blood (Kg) by the density of blood, which is about 1.06 Kg/L [26]. These values for the second and third trimesters were added to the calculated prepregnancy blood volume, resulting in estimates for the second and third trimester blood volumes. A description and the source of data for the model parameters are summarized in Table 1.

**Table 1:** Description and source of data for the model parameters

Data on mother-child pairs from the CHARGE study	Symbol	Units
Bloodspot Hg concentration	*C*_*B*_	ppb
Maternal fish consumption		
Rate during each time step	*I*_*j*_	Kg⋅day^−1^
Time steps	*t*_*j*_	days
Gestational age at birth	*t*_*q*_	days
Blood volume	*V*_*j*_	L
Assumed parameters		
Fraction of MeHg in blood^a^	*f*	-
MeHg elimination Rate^b^	*k*	day^−1^
Estimated parameter		
Fish concentration	*c*	ppb

^a^Sherlock et al., [22]

^b^WHO, [1]

We performed analyses assuming newborn blood spot MeHg concentrations were equivalent to maternal blood MeHg concentrations at the time of birth, and repeated the analyses assuming bloodspots had concentrations 1.5 and 2 times the concentration of the corresponding mother. The ratio of 1.5 and 2 were selected based on previously reported values suggesting that the mercury concentration in cord blood is higher than that of the corresponding mother [27, 28]. We excluded 161 mother-child pairs with missing information pertaining to fish consumption of any type during any time period, leaving 296 mother-child pairs. We further excluded 9 mother-child pairs with missing gestational age data and 13 mother-child pairs with missing maternal weight, leaving a final sample size of 274 mother-child pairs for these analyses.

To estimate cumulative dose during the gestational period, we used the combined fish types MeHg concentration estimate and computed the area under exposure-time curve for each trimester by integrating the pharmacokinetic model with respect to time from conception up until the day of bloodspot collection, t_q_, and summing the cumulative exposure for each trimester to get the cumulative gestational exposure. We also estimated the cumulative exposure over the 2nd and 3rd trimester combined. Cumulative exposure was estimated among the ASD, DD/AtD, and TD groups and the medians were compared using the Kruskal-Wallis Test (due to the non-normal distributions). We also constructed multinomial logistic regression models to look at the association between natural log cumulative MeHg concentration and the risk of ASD and DD (vs. TD). Because values of MeHg concentration included 0, we added 0.5 to all cumulative concentrations prior to taking the natural log. We investigated if the associations were confounded by gender, maternal age (centered at the mean), maternal education, maternal birthplace, child’s race/ethnicity, and payment method for child delivery. We considered a covariate a confounder if it changed the OR for Autism associated with a one unit increase in cumulative mercury on the natural log scale by 10 % or more upon its removal from the model.

## Results

The ASD and TD groups were similar with respect to maternal education, maternal age, child’s race ethnicity, and payment method for child delivery. The ASD group had a higher proportion of males than the DD/AtD and TD groups. As compared with the ASD and TD groups, the DD/AtD group had fewer mothers with a bachelor’s or graduate/professional degree, more mothers born in Mexico, more Latino children, and a higher proportion of deliveries covered by public health insurance (Table 2). The general characteristics were similar between the full data set and the subset with complete fish consumption data. The bloodspot mercury concentration distributions were similar among all diagnostic groups (Table 3).

**Table 2:** General characteristics of each developmental group

	ASD	DD/AtD	TD
*Numbe*r	164	35	58
Gender, *n* (%)			
Males	149 (91 %)	22 (63 %)	46 (79 %)
Females	15 (9 %)	13 (37 %)	12 (21 %)
Maternal education, *n* (%)			
Less than High School or High School			
Degree	24 (15 %)	11 (31 %)	15 (26 %)
Some College or Vocational School	68 (41 %)	14 (43 %)	12 (21 %)
Bachelor’s Degree or			
Graduate/Professional Degree	72 (44 %)	23 (26 %)	31 (53 %)
Average maternal age (years)	31	30	31
Birth place of mother, n (%)			
USA	124 (75 %)	23 (66 %)	44 (76 %)
Outside USA	40 (25 %)	12 (34 %)	14 (24 %)
Child’s race/ethnicity			
White (non-Latino)	78 (47 %)	11 (31 %)	25 (43 %)
Latino	52 (32 %)	17 (49 %)	22 (38 %)
Other	34 (21 %)	7 (20 %)	11 (19 %)
Payment method for delivery, *n* (%)			
Public	31 (19 %)	12 (34 %)	9 (16 %)
Private	133 (81 %)	23 (66 %)	49 (84 %)

**Table 3:** Distribution of bloodspot Hg concentrations (ppb) by developmental group

	ASD	DD/AtD	TD
N	164	35	58
Range	0.07, 40.65	0.90, 15.84	0.18, 22.63
Mean	4.73	4.29	4.73
Median	3.41	3.49	3.48
Geometric Mean	3.34	3.24	3.67
(95 % C.I.)	(2.97, 3.84)	(2.50, 4.19)	(3.02, 4.46)

**Fig. 1:**
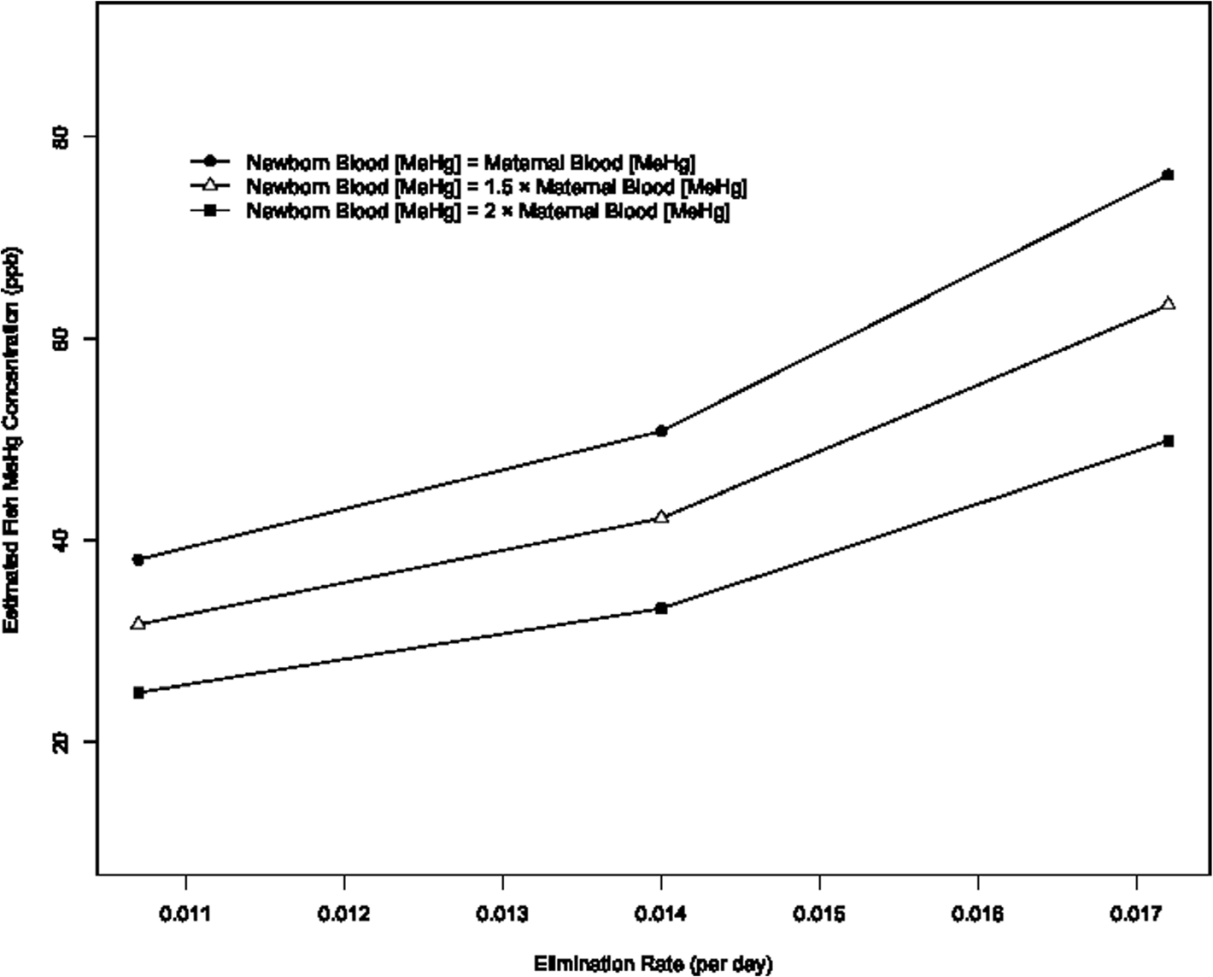
The effect of varying the elimination rate of MeHg from the blood and the assumed relationship between newborn blood MeHg and maternal blood MeHg concentration

The estimated MeHg concentration for tuna, other ocean fish, and fresh fish were 5 (95 % CI: −65,75), 50 (95 % CI: 8,91) and 39 (95 % CI: −29, 106) ppb, respectively. The estimated MeHg concentrations for the combined fish types were similar between the two methods of determining blood volume from maternal weight. If we assumed that maternal newborn blood mercury concentrations were 1.5 times that of the corresponding mothers, the estimated fish concentration was 42 ppb (95 % C.I. 12, 72) when blood volume was based on absolute maternal weight, and 47 ppb (95 % C.I. 15, 78) when blood volume was based on maternal weight gain. Changing the elimination rate by 2 standard deviations leads to about a 10 ppb change in the fish MeHg concentration estimate. The estimated fish MeHg concentration is directly proportional to the ratio between maternal and newborn MeHg concentrations (Fig. 1).

**Fig. 2:**
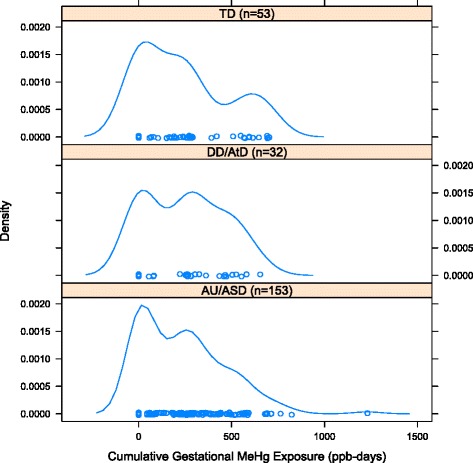
Distribution of cumulative prenatal MeHg exposure in each diagnostic group. All groups have similar distributions, with insignificantly different median concentrations (p = 0.92)

Due to incomplete diagnoses, 36 of the 274 mother-child pairs with complete fish consumption data were excluded from analyses comparing cumulative MeHg exposure among diagnostic groups. The cumulative MeHg distributions over the entire gestational period and over the 2nd and 3rd trimesters, computed using the combined fish types MeHg concentration estimate of 42 ppb, were similar among the three diagnostic groups (Table 4, Fig. 2). The method of assigning blood volume from maternal weight gain resulted in higher cumulative MeHg concentrations versus the method based on absolute weight. Using the Kruskal-Wallis Test, we found that the median cumulative MeHg concentrations were not significantly different among the three diagnostic groups for all measurements of MeHg AUC (p-value raged form 0.91 to 0.98, depending on the comparison). The multinomial logistic regression models yielded null ORs for log cumulative MeHg exposure over the gestational period for ASD vs. TD and DD/AtD vs. TD (Table 5). Models investigating cumulative exposure over the 2nd and 3rd trimesters yielded almost identical results (not shown).

**Table 4:** Pharmacokinetic model-based estimate for cumulative MeHg exposure by diagnostic group

	Area under the curve (ppb)					
	2^nd^ and 3^rd^ trimesters			Gestational period		
	ASD	DD/AtD	TD	ASD	DD/AtD	TD
*Number*	153	32	53	153	32	53
Method 1^a^						
Median	133	140	140	219	262	203
95^th^ %	428	360	408	693	580	680
Range	0 - 545	0 - 378	0 - 443	0 - 1231	0 - 654	0 - 702
Method 2^b^						
Median	144	149	152	241	286	218
95^th^ %	438	388	407	732	680	688
Range	0 - 1395	0 - 487	0 - 523	0 - 1703	0 - 713	0 - 800

^a^Method 1 converts individual maternal body weight to volume of blood based on weight during each time period

^b^Method 2 converts individual maternal body weight to volume of blood based on weight gain during pregnancy

**Table 5:** Association between natural log cumulative prenatal mercury exposure and the risk of autism and developmental delay (vs. Typical Development)^a^

	Unadjusted		Fully adjusted*	
	Log MeHg AUC O.R. (95 % C.I.)	*p*-value	Log MeHg AUC O.R. (95 % C.I.)	*p*-value
ASD	1.01	0.72	1.03	0.47
(vs. TD)	(0.94, 1.10)		(0.95, 1.12)	
DD/AtD	1.02	0.70	1.00	0.94
(vs. TD)	(0.91, 1.14)		(0.89 1.13)	

*Adjusted for: Gender, maternal education, child’s race, maternal birth place, maternal age, and payment method for child delivery

^a^Sample Sizes: Au/ASD = 153, DD/AtD = 32, TD = 53

## Discussion

Our goal was to relate the longitudinal maternally-reported fish intake to the measured concentration of Hg in the neonate’s blood at the time of delivery in order to estimate the average MeHg concentration in fish consumed by mothers, and to estimate the cumulative fetal exposure to MeHg. We found that the ASD, DD/AtD, and TD groups did not have significantly different estimated cumulative mercury distributions, both throughout pregnancy and over the 2nd and 3rd trimester. Our multinomial logistic regression models, adjusted for potentially confounding demographic characteristics, yielded null associations between natural log cumulative MeHg exposure and the risk of autism (vs. typical development) and developmental delay (vs. typical development). Our results do not suggest an association between prenatal cumulative MeHg exposure and the risk of autism or developmental delay.

This is the first investigation of the association between prenatal cumulative MeHg exposure and autism. While several studies have investigated the association between mercury and autism, they have mostly focused on the postnatal and post-diagnostic period [29–33], which limits any discussion of etiology. In contrast, this study estimates exposures that predated the autism diagnoses and that occurred during the period of time when the developing brain is most sensitive to MeHg exposure. This study has several additional advantages. Because we had bloodspot biomarkers and data regarding fish consumption during pregnancy, we were able to estimate the average MeHg concentration of fish consumed by mothers in our study, without assuming that maternal blood MeHg concentrations were stable during pregnancy, or relying on externally published, and potentially inappropriate, fish MeHg concentrations. Additionally we were able to use individual-level data on gestational age and maternal weight (converted to blood volume) to refine the pharmacokinetic model and AUC estimates in order to better classify participants according to estimated prenatal MeHg exposures. We were also able to make comparisons between groups of individuals who have confirmed diagnoses based on standardized psychometric evaluations. While we did not find an association between cumulative mercury exposure and autism, there is evidence suggesting mercury still might have a negative impact on cognitive development; future research should investigate the possible association between cumulative prenatal mercury exposure and measures of cognitive development.

This approach is particularly useful when an estimate of cumulative exposure is desired, but only a single biomarker measurement is available. While cumulative exposure can be estimated from a food questionnaire alone, this typically utilizes concentrations for various foods reported by sources external to the study, and may not be comparable to the concentrations in the food consumed by the study population. Instead of relying on externally reported concentrations estimates, the method presented in this paper estimated dose by relating maternally-reported fish consumption to measured blood concentrations. This is especially pertinent to our study population because in 2001, the FDA began advising women of childbearing age who may become pregnant to avoid eating fish species high in mercury. By essentially calibrating the fish concentrations to the observed biomarker data, the accuracy of the dose estimates may be improved compared to methods relying solely on external concentration estimates. Additionally, MeHg exposures based solely on food frequency questionnaires are overestimated, possibly resulting from the over report of fish consumption [8–10]. If fish consumption was over reported, then our fish mercury concentrations would tend to be underestimated to a similar degree, because the resulting cumulative exposure estimates are calibrated to the observed blood spot concentrations. We should note that this is one of the ways in which our method has advantages over using questionnaire data alone; when estimating cumulative fish concentrations, a systematic tendency to over report fish consumption is counteracted by a reduction in the estimated MeHg dose per serving.

Like most statistical models, our approach relies on some simplifying assumptions. For example, we estimate only a single MeHg fish concentration per fish type, despite that fact that MeHg concentrations can vary widely even within the same species of fish [34]. Our linear regression models estimate the MeHg fish concentration averaged over different participants in a study. We also assume that the pharmacokinetic parameters do not vary across individuals. For example, we were unable to reasonably estimate the ratio for maternal blood mercury concentration versus newborn blood mercury concentration for each specific mother-child pair; instead, we assumed an average ratio and used this value for all mother-child pairs. This reduces the predictive ability of the model (depending on how much the ratios vary around our assumed average ratio). Additionally, we assume that average fish concentration does not depend on the time period, enabling estimates of overall average fish concentration versus having to estimate multiple time-specific concentration estimates.

Our measurements in blood spots were total mercury, which is comprised of both inorganic and organic mercury [4]. Because our concentration estimates are based on the estimated average change in total mercury blood mercury concentration per serving of fish, these estimates will not be influenced by the inorganic component of mercury unless there is a relationship between fish consumption and inorganic mercury exposure. This assumption is reasonable because the mercury in fish is mainly methylmercury (about 90 %) and the ingested inorganic fraction is poorly absorbed across the intestinal tract [35]. Furthermore, in a controlled dosing study, Sherlock et al. found that the inorganic fraction of total mercury in blood was less than 5 % [22]. To ensure that dental amalgams (a source of Hg vapor that readily enters the bloodstream) [4] did not confound our estimated fish concentration, we controlled for the number of maternal dental amalgams at the beginning of pregnancy. Maternal dental amalgams weakly predicted about a 0.5 ppb (p = 0.25) increase in blood mercury concentration, and minimally affected the fish concentration estimate. This strengthens our assumption that the organic mercury concentration estimated in the regression model is not influenced by exposures to inorganic mercury.

Although we had initially planned to use the model to estimate separate mercury concentrations for the individual types of fish, those estimates appeared unreliable. According to the FDA, median tuna MeHg concentrations range from 78 to 560 ppb, depending on the species of tuna [34]. Even if we assume a majority of pregnant mothers avoided species high in MeHg, we would expect our tuna concentration estimate to hover around 78 ppb, which is significantly higher than our estimate of 5 ppb. When tuna alone was included in the regression model, the estimated tuna MeHg concentration was 44 ppb. Adding the other types of fish to the model decree.sed the estimated tuna MeHg concentration to 5 ppb. Because the consumption of tuna is associated with the consumption of other types of fish, the other fish types may have subsumed the contribution that tuna has to blood MeHg concentration when they are included in the model. Additional error could stem from the imprecision associated with recalling specific types and quantities of fish.

As described in the methods, we instead summed the self-reported fish consumption rates across fish types (tuna, other ocean, freshwater). Using a single consumption rate for all fish may reduce the effects of imprecision and potential confounding compared to the analysis using individual fish types. The estimated average fish MeHg concentration for the analysis using combined fish types was 42 ppb, assuming an elimination rate of 0.014 and a ratio of 1.5 between newborn and maternal blood. This estimate is well within the range of MeHg concentrations measured in commercially available fish that are lower in mercury including: catfish, cod, salmon, tilapia, trout, and canned light tuna (Table 6). Our estimated concentration of 42 ppb is lower than the median concentration for canned light tuna (78 ppb; sd = 135 ppb) but higher than that for other popular fish types such as salmon, cod, and tilapia. Our estimated MeHg concentration is also lower than the average concentration in the U.S. fish diet estimated by Groth (86 ppb) [36]; however, there is reason to suspect the distribution of the fish species consumed by mothers in our study differ from that of the overall U.S. population. It is a plausible average fish MeHg concentration if mothers in our study followed guidelines and primarily consumed species with low mercury levels. Indeed, a recent study of pregnant women in the Boston area reported "intake of tuna fish in particular decreased from before pregnancy," though there was confusion among some participants about which seafood species are safer to consume during pregnancy [36].

**Table 6:** Examples of fish with lower levels of mercury [34]. ND = Mercury concentration below detection level (Level of Detection = 10 ppb)

Species	Mercury concentration (ppb)		
	Mean	Median	Std. Dev.
Anchovies	17	14	15
Catfish	25	5	57
Pollock	31	3	89
Salmon (canned)	8	ND	17
Salmon (Fresh/Frozen)	22	15	34
Sardine	13	10	15
Tilapia	13	4	23
Trout (freshwater)	71	25	141
Tuna (canned light)	128	78	135

Although estimating the mercury concentration for all fish types combined mitigated error resulting from imperfect recall of specific fish types, the reliability of this method is still dependent on having useful exposure questionnaire responses regarding the quantity of fish consumed, which are more difficult to obtain as questions address behaviors further back in time. For example, women in our study may not have been able to accurately recall their fish consumption rates in early pregnancy or prior to pregnancy. The use of pharmacokinetic weights ensures that the calibration is most heavily influenced by recent exposure behaviors, but even those are measured imprecisely using questionnaires. In some settings errors in dietary recall could be worse than the repeat reliability of a biomarker, in which case our methods might harm rather than improve the accuracy of exposure assignment compared to using a single biomarker alone. We suspected that might be the case for our analysis based on separate consumption rates for different fish types, which is why we combined the fish types for our final model. Future studies using pharmacokinetic calibration could also employ Bayesian techniques [37] to account for uncertainties in self-reported exposure behaviors, rather than treating them as fixed covariates as we have done here.

To estimate the average MeHg concentration in fish consumed by mothers during pregnancy, we relied on various pharmacokinetic parameters estimated in previous studies. These included the elimination rate of MeHg from the blood, the fraction of ingested mercury present in the blood after absorption across the gastrointestinal tract and distribution throughout the body, and the ratio of MeHg concentration between maternal and newborn blood. While the assumed values of the estimated pharmacokinetic parameters among our study participants likely differ from the true values, we were able to adjust parameters in our models to assess the impact of these assumptions on the estimated MeHg concentration. We found that a dramatic increase (2 standard deviations) in the elimination rate only increased the fish concentration estimate by about 10 ppb. The ratio between maternal and newborn blood MeHg concentration was proportional to the estimated fish MeHg concentration. The variations in our fish MeHg concentration (about 25 to 75 ppb) correspond to fish species that are low in MeHg.

We measured the Area Under the Curve (AUC) of MeHg exposure over the pregnancy period and over the 2nd and 3rd trimesters. This metric was chosen to determine if increasing exposure to MeHg over time is associated with autism. We used the MeHg concentration of the combined fish, 42 ppb, because the estimates produced in this model were robust to varying model assumptions and could be supported by published fish concentrations.

This method can easily be extended to other epidemiologic studies in which there is a biomarker measurement and reliable questionnaire data regarding exposure, given that the single-compartment model is reasonable and that pharmacokinetic parameter estimates are available. While the questionnaire allowed us to estimate the average overall fish MeHg concentration, we could not accurately estimate the concentration for the individual fish types, which may have resulted in more specific individual cumulative dose estimates. Although combining the fish types into a broader category blends the individual concentrations together, this may not lead to a substantial amount of misclassification because the MeHg concentrations vary widely from one fish to the next within a fish type and the levels also overlap extensively among the different fish types, with coefficients of variation often exceeding 100 % (Table 6). Studies utilizing this method may benefit from grouping sources of exposure that have similar concentration distributions, and if possible, into categories that are not correlated with one another. Categories based on externally reported MeHg concentration could also provide a way to validate the model estimates, as the estimated MeHg concentration would be expected to increase across categories with increasingly higher MeHg concentrations, e.g. salmon versus tuna. In cases where there is only one source of exposure, the estimated concentration estimate could be compared to the expected concentration (based on externally reported values) as a way to validate the reported level of exposure. Assuming externally reported values are accurate and the model assumptions are reasonable, lower than expected concentration estimates would suggest a tendency to over report exposures, while higher than expected concentrations would suggest underreporting.

## Conclusions

The toxicokinetic model described in this paper yielded fish MeHg concentration estimates that are consistent with fish species containing lower levels of MeHg. Overall, cumulative MeHg exposure does not appear to elevate the risk of autism or developmental delay. Although the model estimating seafood mercury concentrations relies on several simplifying assumptions and can be prone to error resulting from imperfect recall of seafood consumption, it may have advantages over the use of either single time-point biomarkers or self-reported dietary data alone. This method can easily be extended to other epidemiologic studies in which there is a biomarker measurement and questionnaire data regarding exposure.

## Abbreviations

MeHg: Methylmercury
EEQ: Environmental exposure questionnaire
CHARGE: Childhood autism risks from genetics and the environment]
ASD: Autism spectrum disorder
DD: Developmentally delayed
TD: Typically developing
AtD: Atypically developing
ADI-R: Autism diagnostic interview-revised
SCQ: Social communication questionnaire
ADOS-G: Autism diagnostic observation schedule-generic
MSEL: Mullen scales of early learning
VABS: Vineland adaptive behavior scales
ICP-MS: Inductively coupled plasma mass spectrometry
AUC: Area under the curve
